# Recent Advances in Single-Cell RNA-Sequencing of Primary and Metastatic Clear Cell Renal Cell Carcinoma

**DOI:** 10.3390/cancers15194734

**Published:** 2023-09-26

**Authors:** Adele M. Alchahin, Ioanna Tsea, Ninib Baryawno

**Affiliations:** Childhood Cancer Research Unit, Department of Women’s and Children’s Health, Karolinska Institutet, 10000-19999 Stockholm, Sweden; adele.alchahin@ki.se (A.M.A.); ioanna.tsea@ki.se (I.T.)

**Keywords:** renal cell carcinoma, single-cell RNA-seq, cell-of-origin, tumor microenvironment, stromal cells, immune cells, novel therapies

## Abstract

**Simple Summary:**

In recent years, several therapeutic advances have been made in clear cell renal cell carcinoma (ccRCC) resulting in novel treatment regimens of increased effectiveness. These advances are largely due to breakthroughs in technologies, particularly in transcriptomics, such as single-cell RNA sequencing (scRNA-seq). Using this technology, we have gained a deeper understanding of the biology of ccRCC and revealed various cell populations and their interactions in disease progression. While localized ccRCC patients have shown promising responses to treatment, however, patients with advanced or metastatic disease remain a therapeutic challenge. To address this gap, recent studies have utilized scRNA-seq to investigate both primary and metastatic ccRCC in search of promising therapeutic targets. This review aims to summarize the current state of knowledge in the field, highlight available treatment options and underscore the critical steps needed to improve survival rates, especially for metastatic ccRCC patients.

**Abstract:**

Over the past two decades, significant progress has been made in the treatment of clear cell renal cell carcinoma (ccRCC), with a shift towards adopting new treatment approaches ranging from monotherapy to triple-combination therapy. This progress has been spearheaded by fundamental technological advancements that have allowed a deeper understanding of the various biological components of this cancer. In particular, the rapid commercialization of transcriptomics technologies, such as single-cell RNA-sequencing (scRNA-seq) methodologies, has played a crucial role in accelerating this understanding. Through precise measurements facilitated by these technologies, the research community has successfully identified and characterized diverse tumor, immune, and stromal cell populations, uncovering their interactions and pathways involved in disease progression. In localized ccRCC, patients have shown impressive response rates to treatment. However, despite the emerging findings and new knowledge provided in the field, there are still patients that do not respond to treatment, especially in advanced disease stages. One of the key challenges lies in the limited study of ccRCC metastases compared to localized cases. This knowledge gap may contribute to the relatively low survival rates and response rates observed in patients with metastatic ccRCC. To bridge this gap, we here delve into recent research utilizing scRNA-seq technologies in both primary and metastatic ccRCC. The goal of this review is to shed light on the current state of knowledge in the field, present existing treatment options, and emphasize the crucial steps needed to improve survival rates, particularly in cases of metastatic ccRCC.

## 1. Introduction

Renal cell carcinoma (RCC) encompasses a group of malignant tumors originating from the epithelium of the proximal part of the renal tubules. The most prevalent histological subtype is clear cell RCC (ccRCC), accounting for approximately 75–80% of all RCC cases, followed by papillary RCC (pRCC; 10–15%) and chromophobe RCC (5–10%) [1]. Among RCC patients, about one-third develop bone metastasis, with ccRCC being the most common subtype. Unfortunately, the 5-year survival rate for ccRCC bone metastasis is less than 10%, compared to 75% for non-metastatic ccRCC [2].

ccRCC is often characterized by the loss of chromosome 3p and a second-hit loss-of-function mutation in the *VHL* tumor suppressor gene located on chromosome 3 [3]. Additional chromosomal alterations commonly observed in ccRCC include loss of 14q and gain of 5q. The inactivation of the *VHL* protein leads to an increase in the transcription factor hypoxia-inducible factor (*HIF*), resulting in the transcriptional upregulation of hypoxia-inducible genes, such as vascular endothelial growth factor (*VEGF*) [3,4]. The elevated *VEGF* levels in the tumor microenvironment drive various downstream effects, including enhanced cell proliferation, angiogenesis, migration, and altered metabolism. Besides *VHL*, other frequently mutated genes in ccRCC include *PBRM1*, *BAP1*, *SETD2*, *UTX*, *ARID1a*, and *KDM5a*, which further contribute to the complex genomic landscape of ccRCC [5]. ccRCC tends to metastasize to the liver, lung, bone, brain, pancreas, skin and muscle [6]. One of the most aggressive metastatic sites is the bone. Around 30% of patients with ccRCC develop secondary tumors with a 5-year survival of less than 10% [7,8].

Early interpretations of the cellular landscape of ccRCC were achieved using bulk RNA-sequencing [9,10]. Although a powerful tool, bulk RNA-sequencing measures average gene expression across a population of heterogeneous cells and thus fails to distinguish subtle transcriptional differences or rare populations of cells. Following the emergence and commercialization of single-cell RNA-sequencing (scRNA-seq) a new avenue of unprecedented resolution was opened through which tumor heterogeneity could be unraveled on the individual cell level [11,12,13]. Through the use of single-cell transcriptomics, essential gaps of knowledge are being uncovered along with the rising technological possibilities as the discovery of the cell of origin of ccRCC, immune cell reprogramming post-treatment, and immune and stromal cell characterization of treatment naïve ccRCC patients [14,15,16,17]. Hence, this review aims to shed light on how the use of single-cell transcriptomics technology has assisted recent scientific research in unraveling the tumor microenvironment as well as the way it has supported current clinical trial targets and treatment modifications.

## 2. Methods

The narrative of the review was predetermined to cover the aspect of single-cell technologies. Over a period of four months, searches were made on scientific databases such as PubMed including keywords including “clear cell renal cell carcinoma”, “single-cell RNA sequencing” and “transcriptomics”. For the section describing spatial and transcriptomic evidence, a key inclusion criterion was that the study had used a single-cell and “spatial technology” when describing their findings.

## 3. Unveiling the ccRCC Cell of Origin

Two recent human single-cell transcriptomics studies have provided compelling evidence supporting proximal tubular epithelial cells (PTECs) as the cellular origin of ccRCC. These studies utilized single-cell transcriptomic analysis to compare the transcriptome of captured ccRCC PTECs with single or bulk normal and ccRCC transcriptomes [14,18]. The combined analysis of these studies revealed the expression of several genes, including carbonic anhydrase 9 (*CA9*), vascular cell adhesion molecule-1 (*VCAM1*), solute carrier family 17 member 3 (*SLC17A3*), intercellular adhesion molecule 1 (*ICAM1*), integrin subunit beta 8 (*ITGB8*), alpha kinase 2 (*ALPK2*), and vimentin (*VIM*), in ccRCC PTECs [14,18].

**Figure 1:**
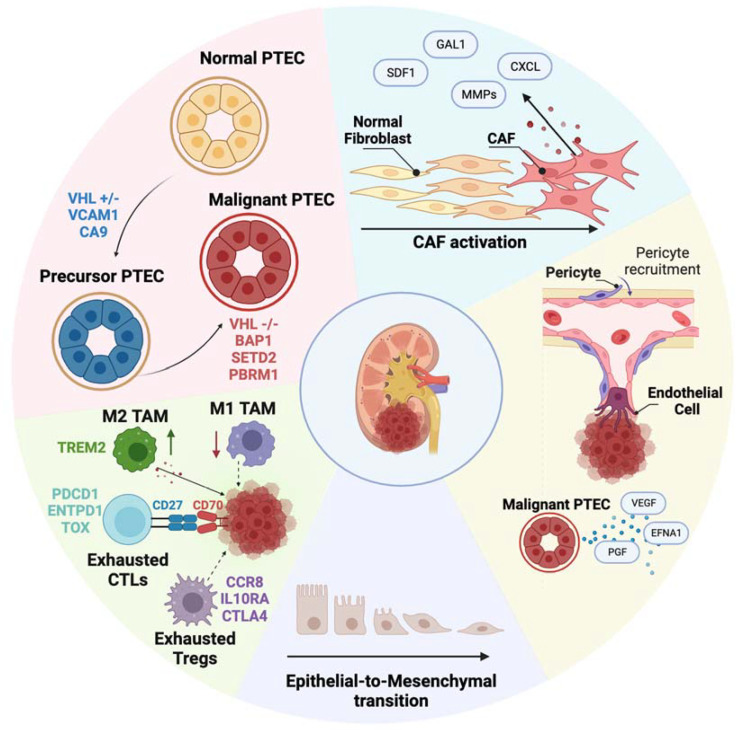
Summary of the biological findings validated with single-cell RNA sequencing studies in primary and metastatic ccRCC. These studies uncover the multilayer complexity of the disease and highlight. (1) The cell of origin of ccRCC as malignant PTECs. Normal PTECs transition towards Precursor PTECs harboring the *VHL^+/−^* mutation and finally to malignant PTECs harboring the *VHL ^−/−^* mutation as well as additional genetic alterations. (2) The progressive dysfunction of the immune cell landscape is characterized by a simultaneous upregulation of dysfunctional M2−like TAMs and downregulation of M1−like TAMs as well as the emergence of terminally exhausted immune cell types including CTLs and Tregs. (3) The EMT transition process and its role in disease progression. (4) The role of non-immune TME cells in promoting angiogenesis and tumor cell invasion. In particular subsets of pro-angiogenic endothelial cells and capillary pericytes have been identified to aid ccRCC disease progression. (5) The role of CAFs in supporting tumor progression and metastasis through the secretion of molecules that drive immunosuppression, extracellular remodeling and EMT. Figure created in Biorender.com (accessed on 15 August 2023).

Of particular interest was the identification of *VCAM1* and *CA9*-positive PTECs in ccRCC patients’ adjacent morphologically normal kidney tissue which were termed precursor PTECs, representing morphologically normal PTECs with *VHL^+/−^* mutation [14]. This finding suggests that identifiable transcriptomic alterations occur following genomic alteration in precursor PTECs, which precede morphological changes in ccRCC development. It supports the proposed concept of a transition from normal to precursor and ultimately malignant PTEC states [14]. A multi-omics study by Muto et al. [19], combining single-nucleus RNA sequencing (snRNA-seq) with single-nucleus ATAC sequencing (snATAC-seq) further investigated precursor PTECs expressing *VCAM1* and *CA9* and showed that they exhibit a transcriptomic similarity to inflamed PTECs characterized by *VCAM1* expression but without *CA9* expression [19]. Inflamed PTECs were defined by the expression of *VCAM1*, *ICAM1*, *CD24*, *CD133*, and *HAVCR1*, and were associated with the response to acute and/or chronic tubular injury. The transcriptomic profile of inflamed PTECs exhibited the strongest similarity to malignant PTECs in ccRCC. This observation suggests an alternative PTEC transition from normal to inflamed, then to precursor, and finally to malignant PTEC states during ccRCC development (Figure 1). The presence of common gene expression patterns in both inflamed and malignant PTECs suggests a potential link between tubular-injury-related inflammation and ccRCC pathogenesis.

## 4. Elucidating the Transcriptomic Identity of Metastasizing Cells

In light of the increasing discoveries on the cell of origin of primary ccRCC, the field quickly proceeded toward the characterization of metastatic ccRCC using single-cell transcriptomic technologies. Indeed, a recent single-cell transcriptomics study analyzing ccRCC primary, locally invasive, and adjacent normal tissues identified enhanced extracellular matrix (ECM) remodeling by malignant PTECs in locally invasive lesions [20]. The findings suggest that while locally invasive ccRCC lesions may result from opportunistic extension into nearby vasculature, the extending malignant PTECs also depend on a supportive ECM. Similarly, metastatic ccRCC progression has been characterized by 17 metastasis-associated gene (MAG) markers identified in a single-cell transcriptomics study involving 121 single-cell samples [21,22]. These cells were captured from parental metastatic sites and patient-derived xenografts of primary and metastatic ccRCC samples [21,22]. The MAGs include chemokines (*CCL20* and *CXCL1*), as well as mitochondrial (*MT-ND3*, *MT-ND4*, and *MT-RNR2*) and cancer markers (*NDUFA5*, *NNMT*, *BHLHE41*, *ALDH1A1*, and *BNIP3*). Expression of these MAG markers correlate with a higher likelihood of ccRCC recurrence. Moreover, a single-cell transcriptomics study comparing treatment-naive primary tumor tissue matched adjacent normal kidney tissue and tumor samples collected from patients with bone metastases deduced a distinct transcriptional signature that is correlated with metastatic potential and patient survival [17]. Another study harnessed the power of multi-omics to highlight *SERPINE2*, a gene in metastatic RCC that could predict metastatic outcomes and further be targeted [23]. These studies provide novel insights into locally invasive and metastatic disease, which remain a therapeutic challenge and lead the field towards their etiology.

One of the major challenges in treating metastatic ccRCC is the significant intratumoral heterogeneity of tumors that is comprised of subclones with diverse genotypes [24]. Hence, recent single-cell studies have attempted to characterize the transcriptional identity of metastatic clones and found two distinct subpopulations able to separately stimulate VEGF- and Epithelial-to-Mesenchymal (EMT) related pathways [25]. Other studies focused on characterizing the process of EMT itself as a driver of tumor metastasis. In particular, a study accomplished to define an EMT metastatic program in ccRCC where they discover cells with an EMT high profile localized in the interface of the tumor and normal environment, which is the leading and migratory margins of a tumor [26].

## 5. Deciphering the Role of the TME in ccRCC Progression

In addition to cancer cells, the TME encompasses non-malignant cell types embedded within an altered ECM. The specific composition of the TME can vary among different tumor types, but it typically includes various cell types such as fibroblasts, adipocytes, neurons, endothelial cells, immune cells, and stem cells, along with secreted molecules like cytokines, chemokines, and growth factors^9^. The advent of innovative techniques like single-cell transcriptomic sequencing has facilitated a deeper understanding and cataloging of this context.

## 6. Immune TME in Primary and Metastatic ccRCC

Several single-cell transcriptomics studies have provided valuable information about immune cell populations captured from primary and metastatic ccRCC tumors. These studies shed light on the transcriptomic profiles of myeloid and lymphoid cell types, states, and their interactions in ccRCC, with particular emphasis on tumor-associated macrophages (TAMs) and CD8+ T cells, both of which play significant roles in tumor progression and evasion. More specifically, the high plasticity of TAM populations was highlighted in ccRCC, spanning a continuum from pro-inflammatory M1-like to anti-inflammatory M2-like states, with intermediate TAM subpopulations based on *HLA-DR* or interferon signaling gene expression levels [16,27]. One study has provided novel insights into the immune cell landscape and correlated its progressive dysfunction with disease stage in ccRCC patients by demonstrating a general shift in TAM states with ccRCC progression, with an increase in dysfunctional M2-like TAMs and a simultaneous decrease in M1-like TAMs [27]. An additional study correlated a *TREM2*-positive TAM population to lower survival in primary ccRCC patients [17,28]. Similarly, single-cell transcriptomic analysis of CD8+ T cells in ccRCC samples reveals a diverse and heterogeneous population, spanning a continuum that progresses to terminally exhausted clonotypes [28,29]. Several studies have unveiled distinct subsets of CD8+ T cells, including naïve, cytotoxic, exhausted, progenitor, and terminally exhausted states [15,27,28,30,31]. The identification of immune inhibitory markers on CD8+ T cells aligns with bulk RNA-seq studies, suggesting potential epigenetic reprogramming leading to exhaustive states through *TOX2* [32,33,34,35]. Within the exhausted CD8+ T cell population, the presence of progenitor and terminally exhausted subpopulations suggests a spectrum of exhausted states that may transition from a progenitor (*TCF7*) to a terminally exhausted (*ENTPD1*) state [15,27,36].

The role of immune cell infiltration in metastatic ccRCC is also gaining more attention as multiple single-cell transcriptomics studies suggest it might be affecting prognosis [27,30]. Two studies in metastatic ccRCC have characterized TAMs to have high expression of both HLA class I and II genes along with *IFI27*, *CTSL*, *CTSS*, *C1QA*, *C1QB*, *SERPING1*, *APOE*, and *PLTP* [27,30]. Moreover, inferred pseudotime trajectory analysis of CD8+ T cells in ccRCC, indicated a higher prevalence of exhausted CD8+ T cells in advanced and metastatic ccRCC compared to normal kidney tissues and peripheral blood [27,28,30,32]. Hence, the potential of immunotherapeutic strategies and immune-related pathways have been increasing as potential directors in cancer therapy care [37]. For instance, PD-1 has been implied to act negatively as an immunoregulatory molecule and to be involved in the regulation of cancer cell immune evasion [38]. However, a research study showed that standard pre-treatment T cell receptor (TCR) clonality could predict clinical response to anti-PD-1 therapy in ccRCC [39], while others observe a considerable variation of TCR clonality across disease stages of ccRCC [27].

While some single-cell studies have provided novel insights into the immune cell landscape of ccRCC, other studies focused on characterizing the complete TME of treatment-naive patients [17] as well as the consequences of ICB therapy in reprogramming the TME [15,31]. In metastatic RCC, one study integrated multi-omics analysis of bulk RNA-sequencing, scRNA-seq, ATAC-seq and 3D high-throughput chromosome conformation capture (Hi-C) to highlight the influence of the TME on the clinical responsiveness towards targeted therapy or immunotherapy [23]. Of particular clinical significance was the demonstration of the capacity of malignant PTECs to drive angiogenesis through the secretion of the *VEGFA*, *PGF* and *EFNA1* ligands and their interactions with receptors on macrophages, fibroblasts and endothelial cells [14,18,31,32]. This evidence indicated by numerous single-cell transcriptomics studies confirmed the existence of interactions between cancer cells and elements of the TME which could represent promising targets and spearheaded the clinical efforts for therapeutic targeting.

## 7. Non-Immune TME in Primary and Metastatic ccRCC

Nevertheless, while the use of antiangiogenic treatments has made VEGF targeting a favorable choice for ccRCC, these therapies often fail to sustain a long-term clinical response in patients. As a result, there has been increasing focus on non-malignant and non-immune stromal cells within the tumor microenvironment. Among these cells, cancer-associated fibroblasts (CAFs) have garnered attention due to their potential immunosuppressive functions within the ccRCC microenvironment. One study revealed that the immunosuppressive behavior mediated by CAFs is attributed to the secretion of Galectin-1 (*Gal1*) which induces apoptosis in cytotoxic CD8+ T cells in recurrent ccRCC [40]. It is suggested that the recruitment of CAFs into the ccRCC microenvironment occurs through interactions with malignant PTECs that upregulate *COL20A1*, *COL28A1*, and *TGFB1* [20]. Indeed, both Alchahin et al. and Shi et al. identified CAF-mediated extracellular matrix remodeling, which was associated with an increased gene signature for the EMT pathway in primary and locally invasive ccRCC, respectively [17,20]. Therefore, in both primary and recurrent ccRCC, the infiltration of CAFs should be considered as an additional critical cell type driving tumor progression and immunosuppression.

Endothelial cells, responsible for blood vessel formation and pericytes that surround and stabilize blood vessels exhibit distinct subpopulations within the ccRCC TME as revealed by scRNA-seq analysis. Multiple single-cell studies in primary ccRCC have identified endothelial cell subsets with differential expression of genes involved in angiogenesis, vascular stability, and immune modulation. These subsets were associated with immune cell infiltration, angiogenesis, and therapy response, highlighting their functional specialization and impact on the ccRCC TME [14,17,41]. Alchahin et al. highlighted the enrichment of pro-angiogenic capillary pericytes in treatment-naive ccRCC coupled with the reduction in vascular smooth muscle cells, known to maintain blood vessel integrity, thus showing the active remodeling of the TME in ccRCC progression [17].

## 8. Treatment of ccRCC and ccRCC Metastasis

A localized ccRCC tumor is still resected through partial or radical nephrectomy as the standard of care [42]. Even if there are signs of a simultaneous formation of micrometastasis, surgical resection is proven to be efficient in preventing the early steps of metastasis [43]. Although over the past two decades the treatment approaches have changed [44], particularly in metastatic ccRCC, the ongoing advancements in modern technology will continue to provide insights into the complexities of cancer and its metastasis, which may inspire further changes in treatment approaches. For metastatic disease, the treatment now comprises multiple targets that have been developed to block the activity in signaling pathways of mammalian target of rapamycin (*mTOR*), vascular endothelial growth factor (*VEGF*) pathways and platelet-derived growth factor (*PDGF*). They have demonstrated an involvement in angiogenesis and metastasis further promoting the development and progression of ccRCC [45,46,47,48,49]. Despite the efficacy of the initial treatments used, the median time for the patients to obtain drug resistance is around 6–15 months, differing based on therapeutic schedule and intratumor heterogeneity (ITH) [50,51]. Hence, further research is required to overcome this therapeutic hurdle.

The somatic *VHL* mutation known in ccRCC was one of the earlier discoveries [52] that resulted in the idea of preventing tumor angiogenesis via targeting the *VEGF* pathway, including its receptors, where mainly *VEGFR2* is targeted [53]. ccRCC tumors are a group of epithelial tumors that present with elevated expression of *VEGFA* and are therefore an understandable target of the disease [54]. With scRNA-seq technology, it was possible to identify cell populations in ccRCC that normally express *VEGF*-related pathway genes including subsets of endothelial cells [14,17]. Several studies have shown the binding of *VEGF-VEGFR2* to significantly enhance tumor development, contributing to its progression and expansion [55,56,57]. One of the first agents developed for targeting and inhibiting both *VEGF* and *PDGFR* in metastatic RCC was sunitinib, a tyrosine kinase inhibitor (TKI) which at the time presented significant progression-free survival of 11 months in comparison with 5 months with the earlier broadly used interferon alfa [58,59]. Along with the targeting of angiogenesis, there is a particular interest in inhibiting the *mTOR* pathway in ccRCC because of the known involvement of the regulatory effects on *HIF2a* production, but also its role in regulating cell proliferation and survival processes [60]. It is the *HIF2a* that is involved in the upregulation of *VEGF* further promoting angiogenesis [60]. When dual therapy was introduced by combining an *mTOR* inhibitor everolimus with levantinib (*VEGF* inhibitor), it demonstrated prolonged progression-free survival [61].

As dual therapy emerged, showing promising effects in patients with advanced ccRCC, the evolving single-cell transcriptomic knowledge of immune cell infiltration and dysfunction harbored the new therapeutic era [14,17,27,62]. The discovery that ccRCC tumors are highly infiltrated by T cells with the exhaustive phenotype [9] led to modified therapeutic approaches by adding immune checkpoint blockade (ICB) as an additional alternative in combination with antiangiogenic drugs [9,63,64,65]. When comparing monotherapy of antiangiogenic agents to a combination with ICB in clinical trials, a significantly improved overall survival was observed [61,64,65,66,67]. The response rates of combination therapy ranged between 42–71% [61,64,65,66,67]. Immunotherapy has therefore surpassed clinical expectations where both monotherapies of anti-*PD-1* agents and dual use of *PD-1* (programmed cell death protein 1) and *CTLA-4* (cytotoxic T lymphocyte–associated protein 4) inhibition show effective therapy responses of a median of 24 months in patients diagnosed with non-ccRCC and metastatic ccRCC [66,68,69]. These novel immunotherapies are capable of reviving T cell exhaustion to re-initiate tumor-killing effects [70]. Recent utilization of scRNA-seq has effectively uncovered the existence of a highly immunosuppressive microenvironment in both primary and metastatic ccRCC and provides a basis for therapeutic targeting using the aforementioned approaches and combinations [17]. Furthermore, combining immunotherapy with antiangiogenic agents has significantly improved progression-free survival indicating the important role of the TME and how it can be manipulated for new treatment strategies in metastatic ccRCC [64,65,71]. In patients with metastatic ccRCC that had previously been treated with antiangiogenic agents, the *PD-1* inhibitor nivolumab showed better overall survival compared to everolimus [68,72].

Despite promising and improved treatment results, patients with metastatic ccRCC may become unmanageable and the disease may recur. The latest guidelines specified by the European Association of Urology (Arnhem, The Netherlands) [42] concluded that partial nephrectomy remains a superior approach when the disease is localized. However, in metastatic ccRCC, surgical resection is not recommended as inhibition of *VEGFR* and *PDGFR* with sunitinib did not present worse outcomes compared with nephrectomy [73].

Following the idea of multiple targeting for metastatic disease, the latest therapeutic approach involves the triple combination of nivolumab (anti-*PD-1*), ipilimumab (anti-*CTLA-4*) and cabozantinib (TKI and VEGF inhibitor) covering both immune infiltration and known pathways involved in ccRCC progression. Interestingly, it demonstrated clinical efficiency in advanced RCC patients that are treatment naïve [74,75]. Nonetheless, as the results are encouraging, clinical analyses are being assessed. These, and more trials, will shape new therapeutic strategies after retrospective data representation in advanced ccRCC (Table 1). In the ongoing effort to manage advanced ccRCC, the question being explored is whether targeting multiple factors simultaneously offers improved treatment outcomes, as multiple cellular compartments and processes are simultaneously involved in cancer progression [14,17,27,30,62].

## 9. Future Perspectives

Early treatment strategies already distinguished the importance of immunotherapy by T cell receptor proliferation cytokine IL-2 and interferon a2b [78,79]. Today, the recent standard of care is to use immune checkpoint inhibitors which have changed the paradigm of ccRCC therapy [64,80]. Despite these successful treatment strategies, a subset of renal cancer patients still do not respond to treatment, and those who do eventually progress [81,82]. The aforementioned single-cell studies have demonstrated a novel understanding of the cellular landscape of ccRCC. However, by also focusing scRNA-seq on T cells using 5′-sequencing and recombined V(D)J region of T cell receptor, researchers show in ccRCC that tumor-infiltrating T cells harbor a different expression and transcriptional pattern when it is compared to the normal renal tissue and peripheral blood [28], indicating a transcriptomic heterogeneity. Moreover, a single-cell study in melanoma achieved to connect a subset of melanoma-infiltrating lymphocytes to certain antigens of T cell receptors, implying that the expression level of intratumoral CD8+ T cells may be controlled by particular tumor phenotypes [83]. Similarly, in ccRCC, cells existing in a CD8+ T cell receptor (TCR) clonotype were shown to be controlled by their level of exhaustion [26]. This restriction of clonotypes based on phenotype may not depend on environmental factors, but instead on chronological mutations as individual tumors carry clonotypes through different states [84,85,86]. Thus, when cells infiltrate a tumor and undergo changes from an active to a dysfunctional state, they stay in the tissue depending on the phenotype of the tumor tissue they reside in [26].

Manipulating the adaptive immune responses by using ICB has shown us improved survival [65,68], emphasizing the role of the immune microenvironment in ccRCC. Hence, to better understand the architecture, tumor-infiltrating immune cells, immunotherapy and the T cell receptor immune cell atlas, advanced single-cell technologies need to be applied. The single-cell technologies have revealed and confirmed to us the rich tumor microenvironment, including the immune microenvironment [14,17,21,87,88]. The generation of gene signatures deciphering the roles of certain immune subsets in cancer has functioned as a tool in guiding clinicians to select patients, calculating the probability of benefiting from immunotherapy and characterizing clinically relevant subpopulations. Immunotherapy has reached the status of treatment stamina when treating treatment-naïve and advanced ccRCC [64,89].

The massive data collection and production from single-cell studies generate enormous information and hypotheses to test. The comprehensive output has been able to confirm that the current suggested triple therapy may work by proving the pathways and gene expressions that are augmented in primary and metastatic ccRCC as *CTLA-4*, *PD-1*, *VEGFR2* and *MET* [17,74]. In theory, this combination targets four principal aspects influencing ccRCC disease development; immunosuppression, angiogenesis (vascular remodeling) and MET overexpression [17].

With explorative science, new knowledge will be provided, interpreted, and tested. Equally, as single-cell technologies have computationally and quantitatively mapped the TME [14,17,21,87,88], clinical trials are pursuing to evaluate the findings. An example is the recent research emphasizing the *CD70-CD27* axis in primary ccRCC development as a potential target [17]. The finding supports clinical trials that are currently testing agents targeting this axis by engineering CAR-T cells for instance, as *CD27* was expressed by exhausted cytotoxic T cells [17]. This approach is now being tested in clinical trials [76] (NCT05468190, NCT05420519, see Table 1). Hence, single-cell technologies are able to generate massive amounts of information which can subsequently be used to provide solid facts to support clinical trials.

Nevertheless, while scRNA-seq technology has become a state-of-the-art approach for unraveling the heterogeneity and complexity of different cell types, it has also brought to light certain methodological challenges, such as the ”artificial transcriptional stress responses”. In particular, the process of single-cell isolation has been found to trigger the expression of stress-related genes, which in turn can lead to artificial changes in cell transcription patterns [90,91]. Another challenge in scRNA-seq is dealing with variation between different datasets [92]. Data collected at different times or using different sequencing platforms can have significant batch effects [93]. While often not biologically meaningful, these batch effects can disrupt patterns in gene expression and potentially lead to incorrect conclusions. Therefore, it is crucial to correct these batch effects during analysis of single-cell transcriptomics studies. To this end, several algorithms have been proposed to correct batch effects although these methods can be computationally intensive and often require significant amounts of memory and time [94,95].

Perhaps the most significant limitation of scRNA-seq methods is the loss of histological information through the need to dissociate tissue into single-cell suspensions. Hence, novel spatial biological technologies have been emerging rapidly as powerful tools to add the additional layer of spatial information that single-cell technologies are currently missing. A recent study in ccRCC combined the use of single-cell transcriptomics with barcode-based spatial transcriptomics to study the effect of the MC5 lncRNA signature on immunotherapy response and the TME [96]. Two studies combined spatial transcriptomics with scRNA-seq to reveal cell types within the TME of ccRCC that correlate with ICB resistance in ccRCC patients [97,98]. An intriguing example of the power of multi-omics is offered by Wu et al. [99], where single-nucleus transcriptomics, epigenomics, and spatial transcriptomics are employed to identify a novel tumor cell signature correlating with reduced survival in ccRCC patients [99].

In conclusion, the paradigm shift in ccRCC disease progression has been enhanced in the past two decades in terms of biological and therapeutic understanding. As the survival of primary disease is satisfactory, the challenges and future work lie in tackling the metastatic outcome. The research community of ccRCC has a broad and huge data output of hypotheses with computational rationale and strength to test. The next steps are to envision the disease by combining multi-omics data to better understand the cellular profiles and their biological communication patterns with spatial and imaging techniques. This is where the fundamental efforts need to be put on, in parallel with the ongoing clinical trials to understand therapy responses.

## 10. Conclusions

ccRCC tumors are known for their hypoxic, immunogenic, and angiogenic characteristics. To fully comprehend ccRCC, it is essential to investigate these features not only within tumor cells but also in immune and non-immune stromal cells that infiltrate the ccRCC TME. Recent advancements in single-cell transcriptomics applied to primary and metastatic ccRCC tumor samples have significantly enriched our understanding of the diverse cell types and states present in ccRCC.

The discovery of PTECs as the ccRCC cell of origin as well as their inflamed state raises the possibility of an alternative transcriptomic pathway in ccRCC development. Moreover, with our increasing understanding of the transcriptomic identity of metastasizing clones, new therapeutic avenues are being unlocked for metastatic ccRCC. Multiple studies have provided evidence on the dysfunctional interactions of M2-like TAMs and exhausted CD8+ T cells, which may contribute significantly to the resistance observed in available immune checkpoint therapies.

Recent findings on immunosuppressive, angiogenic, and extracellular matrix remodeling activities by CAFs and pericytes as well as endothelial cells suggest that stromal cells play an additional elusive role in primary and metastatic ccRCC. As a result, a comprehensive understanding of PTECs, immune cells, and stromal cell types and states within the ccRCC microenvironment is shedding light on tumor progression and evasion across different stages of ccRCC. Emerging integrative approaches to single-cell technologies overcome the current limitations of the technique and pave the way towards new discoveries that will drive future clinical management, therapeutics, and prognostics for both primary and metastatic ccRCC cases.

## Figures and Tables

**Table 1:** Single-cell RNA sequencing studies that have supportive evidence of the targets in clinical trials of ccRCC and metastatic ccRCC.

Clinical Trials in ccRCC and Metastatic ccRCC	Target Involved in Trial	Single-Cell RNA Seq Studies Supporting the Trial
**NCT05468190**	CD70	[17,76]
**NCT05420519**		
**NCT00944905**		
**NCT03905889**	CDK4/6	[11]
**NCT03945773**	Combination therapy with VEGF	[14,16]
**NCT03473730**	CD38	[16]
**NCT03987698**	PD-1	[20,27,39,65,68]
**NCT05239728**		
**NCT04518046**		
**NCT03729245**		
**NCT03937219**		
**NCT04518046**	CTLA-4, PD-1, MET	[17,27]
**NCT03937219**		
**NCT04691375**	TREM2	[17,30,77]
**NCT05103722**	IL-6	[25]
**NCT04338269**	RTK, PD-1, CTLA-4, TKI c-MET, VEGFR2	[17]
**NCT03141177**		
